# Registered Nurses’ Moral Distress and Coping Strategies in Adult Tertiary Care: A Scoping Review

**DOI:** 10.1155/jonm/9060569

**Published:** 2026-11-15

**Authors:** Hanan Alfaifi, Michael Brown, Clare McVeigh, Lynne Marsh

**Affiliations:** ^1^ School of Nursing and Midwifery Queen’s University Belfast Belfast UK qub.ac.uk; ^2^ Nursing College Najran University Najran Saudi Arabia nu.edu.sa

## Abstract

**Aim:**

To explore registered nurses’ experiences of moral distress, coping strategies, and organisational responses within adult tertiary care settings.

**Background:**

Moral distress presents significant challenges for registered nurses, yet evidence concerning coping strategies and organisational responses remains fragmented.

**Evaluation:**

A scoping review was conducted following Joanna Briggs Institute methodology and PRISMA‐ScR guidelines. MEDLINE, CINAHL, EMBASE, and PsycINFO were searched for English‐language studies published from 2015 onwards. Following an updated search in October 2026, 25 studies met the inclusion criteria.

**Key Issue(s):**

Moral distress was frequently associated with organisational constraints, including inadequate staffing, limited decision‐making authority, and insufficient leadership support, alongside interpersonal conflict and ethically challenging situations. Nurses reported emotional exhaustion, reduced job satisfaction, and intentions to leave. Coping strategies included emotional distancing, resilience, peer support, and moral reasoning, while evidence of structured organisational interventions was limited.

**Conclusion(s):**

Moral distress among registered nurses in adult tertiary care is shaped by interconnected organisational, interpersonal, and ethical conditions. The limited intervention‐focused evidence highlights a need for further research into organisational approaches to addressing moral distress.

**Implications for Nursing Management:**

Nursing managers should consider strengthening ethical practice environments through supportive leadership, improved communication, accessible support mechanisms, and opportunities for reflective practice. Further evaluation is needed to establish the effectiveness of organisational interventions.

## 1. Introduction

Moral distress is a significant and persistent challenge for registered nurses working in adult tertiary care settings. Although many healthcare professionals experience moral distress, research indicates that nurses report higher levels than other staff groups due to increasing expectations and the growing complexity of patient care [1–3]. Moral distress occurs when healthcare professionals recognise the ethically appropriate course of action but are unable to act on it because of institutional constraints [4]. In nursing, where moral and professional values are central to practice, such constraints can lead to profound emotional and professional conflict [4].

Moral distress is often triggered by environments characterised by inadequate patient care, prolonged suffering, underqualified staff, and limited administrative support related to workloads, working hours, and employment conditions [4]. Nurses working in tertiary care frequently report feelings of frustration, grief, and powerlessness, which contribute to a sense of invisibility and disconnection from their work [5]. These experiences may lead to insecurity, loss of professional purpose, and eventual burnout [6]. Moral distress also affects nurses’ perceptions of their ability to provide high‐quality care. Some report taking sick leave when they feel ignored in treatment decisions, whereas others decline to undertake certain procedures or withdraw from patient care when excluded from decision‐making processes [7]. It may also diminish self‐esteem and confidence, contributing to self‐blame [8] and social withdrawal [9].

Studies have highlighted the far‐reaching personal and professional consequences of moral distress. Many nurses describe feeling exhausted, isolated, and permanently changed, with some believing that leaving their workplace is the only way to recover [10, 11]. Over time, repeated exposure to moral distress can desensitise nurses, affect their relationships with colleagues and families, and reduce overall well‐being. The psychological strain associated with moral distress often extends beyond the workplace, with many nurses reporting difficulty disengaging from unresolved situations and carrying feelings of inadequacy home with them [11].

Addressing moral distress requires a comprehensive approach that considers both individual and organisational strategies. Strong leadership, ethics education, reflective practice opportunities, and supportive workplace structures have been shown to mitigate its effects [12]. Nurses also rely on critical thinking, cognitive flexibility, and peer support to navigate morally complex situations [13]. Despite growing recognition of moral distress among nurses, evidence remains fragmented regarding how nurses cope with these experiences and how organisational structures and support mechanisms assist them in managing moral distress. Given this variability and the breadth of existing research, a scoping review was appropriate to map current evidence, clarify key concepts, and identify gaps requiring further investigation. This review aimed to explore how registered nurses in adult tertiary care experience and respond to moral distress, with particular focus on the coping strategies they adopt and the organisational roles and functions that support nurses’ responses to moral distress. Recent reviews have examined moral distress from different perspectives, including nursing leadership [14], evidence synthesised across existing reviews [15], and interventions aimed at addressing moral distress [16]. In contrast, the present review brings together primary empirical evidence on registered nurses’ experiences of moral distress, coping strategies, and organisational responses specifically within adult tertiary care settings. This integrated focus provides a context‐specific understanding of moral distress and the organisational approaches reported in the literature.

## 2. Materials and Methods

### 2.1. Design

This review followed the Joanna Briggs Institute methodology for scoping reviews [17] and adhered to the EQUATOR‐endorsed PRISMA‐ScR checklist [18]. The review question was developed using the population–concept–context (PCC) framework recommended by JBI. The review question was: What is known about registered nurses’ experiences of and coping with moral distress in adult tertiary care settings, including the organisational roles and functions that support nurses in managing moral distress. The PCC framework also informed the eligibility criteria presented in Table 1. The review protocol was deposited in Queen’s University Belfast’s PURE institutional repository.

**TABLE 1:** Eligibility criteria based on the population–concept–context (PCC) framework.

	Inclusion criteria	Exclusion criteria
Population	Registered nurses working in adult tertiary‐care hospital environments	Nursing students, faculty nurses, or healthcare professionals who are not RNs.
Concept	Studies that explicitly examine moral distress among registered nurses, including experiences and contributing factors, coping strategies, and/or organisational responses, roles, or support related to addressing or managing moral distress.	Studies that do not explicitly address moral distress as a central or significant focus or do not provide findings relevant to the review concept.
Context	Adult tertiary‐care hospital settings (including emergency departments, critical care units, and other specialised hospital units).	Paediatric, maternity, primary care, or community‐based settings.
Types of sources	Empirical research (qualitative, quantitative, or mixed methods designs). Peer‐reviewed journal articles. Publications in English	Opinion pieces, commentaries, conference abstracts, and non‐English publications.

### 2.2. Search Strategy

A comprehensive search strategy was conducted following a multi‐step process guided by the JBI Manual for Scoping Reviews (Table 2). To ensure the inclusion of the most contemporary evidence relevant to current practice contexts, the review was limited to studies published from 2015 onwards. As highlighted by Huo et al. [19], healthcare environments are dynamic and continually evolving, underscoring the importance of drawing on recent evidence that reflects changing policies and practices.

**TABLE 2:** Search strategy.

Step	Process
Initial search	An initial search was undertaken to derive additional keywords by analysing the titles and abstracts of previous research and reviews on moral distress
Keyword refinement	Both the original and additional keywords were adjusted and utilised in the chosen databases to achieve a thorough search strategy
Database search	Databases used (MEDLINE, CINAHL, EMBASE, and PsycINFO) using high‐precision keyword and Boolean operator strategies
Reference list screening	The reference lists of included studies were reviewed following full‐text screening; however, no additional eligible studies were identified

This review included studies published in English, as it remains the dominant language of scientific publishing and nursing research. Given that English‐language publications often encompass research from a wide range of geographical and cultural settings, the review still captured a broad global perspective on moral distress among registered nurses. To ensure that the included studies had undergone scholarly scrutiny, the review was restricted to peer‐reviewed publications. The search strategy incorporated synonyms, singular and plural forms, and relevant subject headings. In addition, a precise combination of MeSH terms and keywords was applied to maximise the retrieval of relevant studies and to strengthen the comprehensiveness of the evidence base [20].

The search results were combined using the Boolean operators ‘OR’ and ‘AND’ [21]. Four electronic databases were accessed via EBSCOhost: MEDLINE, CINAHL, EMBASE, and PsycINFO [22]. Additional manual searches were conducted, including grey literature sources such as Google Scholar, OpenGrey, and Policy Commons. Google Scholar provided access to a wide range of peer‐reviewed articles and grey literature; OpenGrey indexed reports and theses; and Policy Commons offered policy documents from research institutions and government bodies. These sources were explored to find further relevant studies; however, no additional eligible records were retrieved. Consultation with a subject librarian supported the development of a comprehensive search strategy and optimisation of database searches. The search keywords used in this review included Moral distress, Moral conflict, Moral dilemma, Moral injury, Ethical distress, registered nurses, Nurs∗, and Coping. Although the inclusion criteria allowed for studies involving newly graduated registered nurses, no eligible NGRN studies were identified during the search.

### 2.3. Inclusion and Exclusion Criteria

Eligibility criteria based on the PCC framework are shown in Table 1.

### 2.4. Search Outcome

A total of 4971 records were retrieved from the database searches. Following the removal of 2648 duplicates, 2323 records remained for title and abstract screening, of which 2252 were excluded. Seventy‐one reports were sought for retrieval, of which four could not be accessed. Of the 67 full‐text articles assessed, 42 were excluded for not meeting the eligibility criteria. Ultimately, 25 studies met the inclusion criteria following the updated search conducted in October 2026.

Studies excluded at the title and abstract screening stage either failed to meet the inclusion criteria or met one or more exclusion criteria. Common reasons for exclusion included a focus on populations outside the scope of this review (e.g., psychiatric nurses, physicians, or other healthcare professionals), studies conducted in nontertiary care settings (e.g., paediatric or home care), and publications that did not constitute empirical research.

Studies with ambiguous abstracts or potential relevance were advanced to full‐text screening for further eligibility assessment. Full‐text articles were excluded for reasons including inappropriate populations, nonempirical designs, unsuitable clinical settings, or insufficient focus on moral distress (e.g., studies limited to measuring distress levels). The screening process is presented in Figure 1.

**FIGURE 1:**
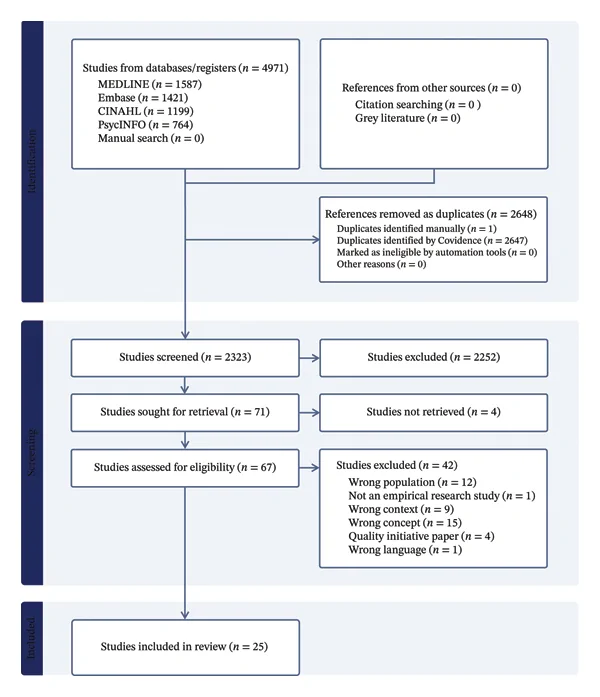
PRISMA‐ScR illustrating the search results.

### 2.5. Data Abstraction

Data abstraction was undertaken using a structured form developed in accordance with JBI methodology for scoping reviews [17]. The form captured key study details, including author(s), year of publication, country, aims, study design, and findings relevant to moral distress. Only data relevant to the review objectives were charted. HA independently abstracted the data, and MB and CM reviewed the abstraction process. The extracted data were then organised to facilitate synthesis in relation to the review objectives. No authors were contacted, as all necessary data were available in the published studies.

### 2.6. Synthesis

Data synthesis was primarily conducted using Braun and Clarke’s [23] thematic analysis approach to identify and organise patterns across the qualitative findings of the included studies. Themes were developed to capture nurses’ experiences of moral distress, coping strategies, and organisational responses. Quantitative findings were subsequently incorporated into the narrative synthesis to complement the identified themes, with differences in study designs considered during interpretation.

## 3. Results

### 3.1. Characteristics of Included Studies

A total of 25 studies met the inclusion criteria. The studies were conducted across multiple countries and employed qualitative, quantitative, and mixed‐methods designs. Collectively, they included 822 participants, comprising registered nurses and nurse leaders working in adult tertiary care settings. Table 3 summarises the characteristics and key findings of the included studies, including findings related to experiences of moral distress, coping strategies, and organisational responses.

**TABLE 3:** Characteristics and key findings of included studies.

Author & year	Country	Aim	Sample	Study design	Key findings
Vilela et al. [24]	Brazil	To understand ethics and moral distress expressions in intensive care nursing practice.	12 RNs	Qualitative: interviews	Ethics in nursing practice are influenced by values, virtues, and relationships. RNs face moral distress due to imbalances in power relations, lack of autonomy, and poor communication.
Hosseinpour & Keshmiri [25]	Iran	To investigate the frequency and intensity of RNs’ moral distress, explore experiences, and develop a conceptual model of risk factors.	180 RNs in the quantitative phase, 20 RNs in the qualitative phase.	Mixed methods: survey, interviews	The frequency and intensity of moral distress were significantly associated with nurses’ roles, patient conditions, and experiences of working with unsafe colleagues.
Pavlish et al. [26]	USA	To explore nurse leaders’ experiences working in ethically difficult situations and helping RNs cope with moral distress.	Data collected from 100 nurse leaders in various positions	Qualitative: open‐ended questions	Identified key strategies to mitigate moral distress, including open communication, emotional support, and ethical training.
Arends et al. [7]	The Netherlands	To explore moral distress among RNs involved in life‐prolonging treatments for patients with short life expectancy.	23 RNs	Qualitative: interviews	Life‐prolonging treatment and lack of involvement in the decision‐making process about the patient′s treatment contributed to experiencing moral distress.
Clark et al. [27]	USA	To explore how resilience, job satisfaction, and moral distress affect workplace engagement among emergency department RNs.	15 RNs	Qualitative: interviews	Participants experiencing moral distress were disengaged from their workplace due to the inability to commit to work responsibilities, taking on extra shifts on their days off, and absent participation in social activities.
Meziane et al. [28]	Canada	To evaluate the effectiveness of a reflective practice intervention on the moral distress of RNs providing end‐of‐life care in acute care units.	16 RNs	Quantitative pilot study of reflective practice intervention	RNs noticed changes in their practice. The results did not show a significant difference in the mean of the pre‐ and postintervention moral distress.
Langley et al. [8]	South Africa	To explore and describe the experiences of moral distress among intensive care unit (ICU) RNs.	33 RNs	Qualitative: open‐ended questions, and focus groups	RNs reported significant distress due to perceived incompetence among colleagues, inadequate staffing, poor communication, and challenging end‐of‐life decisions. This distress was compounded by a lack of managerial and emotional support.
Miller et al. [29]	USA	To explore how unit‐based critical care nurse leaders navigate and address moral distress within their staff.	5 nurse leaders	Qualitative: interviews	The most common approach to addressing moral distress was advocating for and supporting staff. Additional strategies included improving communication, providing emotional support and opportunities to decompress, and maintaining managerial presence.
Tong et al. [30]	USA	To explore ICU RNs’ experiences of moral distress when caring for patients on comfort measures and interacting with families during end‐of‐life care.	79 RNs	Qualitative survey with open‐ended questions	62% of RNs reported experiencing moral distress when providing comfort measures only. Feeling well‐prepared to answer family questions was associated with less moral distress.
Wolf et al. [31]	USA	To explore the nature of moral distress as it is experienced and described by emergency RNs.	17 RNs	Qualitative: focus groups	Moral distress primarily stemmed from conflicts between administrators’ and nurses’ role expectations, with participants using adaptive coping strategies such as exercise, counselling, debriefing, and stress management.
Asadi et al. [32]	Iran	To describe the experiences of ICU RNs regarding ethical decision‐making.	14 RNs	Qualitative: interviews	ICU RNs experience significant moral dilemmas, often feeling torn between professional responsibilities and organisational constraints.
Esmaeili et al. [33]	Iran	To investigate the coping strategies of ICU RNs for reducing moral distress.	17 RNs	Qualitative: interviews	Two categories of coping strategies were identified: desirable (compensation and rejuvenation) and less desirable (indifference over time, escape, and concealment). RNs attempted to resolve ethical issues through discussion but resorted to various coping mechanisms when unsuccessful.
Kiziltepe et al. [9]	Turkey	To explore the experiences of intensive care RNs related to moral distress	18 RNs	Qualitative: focus groups	RNs often encountered moral distress, though many were unfamiliar with the term itself. They linked it to providing futile care, managing family demands, and working in resource‐limited hospital settings. Coping responses varied, ranging from active stress‐relief strategies to emotional withdrawal or avoidance.
Forozeiya et al. [13]	Canada	To describe intensive care RNs ’ experiences of coping with moral distress.	7 RNs	Qualitative: interviews	The study identified coping strategies such as seeking social support, which helped RNs move forward and engage meaningfully with patients. However, failure to cope effectively led to moral disengagement.
Atli Özbaş et al. [34]	Turkey	To explore the sources of moral distress in oncology RNs.	14 RNs	Qualitative: interviews	Failure to provide holistic care, futile treatments, and being unable to advocate for patients are reasons for moral distress.
Lievrouw et al. [35]	Belgium	To explore variations in coping with moral distress among physicians and RNs.	18 RNs	Qualitative: interviews	RNs primarily relied on emotional and experiential coping strategies to manage moral distress, focusing on feelings and experiences rather than rational approaches.
Choe et al. [36]	South Korea	To explore and understand moral distress as experienced by critical care RNs.	14 RNs	Qualitative: interviews	RNs experienced moral distress due to limited autonomy, ethical insensitivity, and conflicts with physicians and institutional policies regarding treatment decisions.
Demir et al. [11]	Sweden	To describe RNs’ experiences of moral distress and how it affects daily work in surgical care.	12 RNs	Qualitative: interviews	In surgical settings, moral distress stemmed from staff shortages and complex patient needs, with heavy workloads and teamwork demands compromising nurse well‐being and patient safety.
Ntseke et al. [37]	South Africa	To explore and describe factors contributing to moral distress experienced by critical care RNs executing Don’t Resuscitate orders.	14 RNs	Qualitative: interviews	RNs experienced moral distress when implementing Don’t Resuscitate orders, as the process conflicted with their values and was intensified by poor communication and unclear guidelines.
Mehlis et al. [38]	Germany	To investigate the prevalence and intensity of moral distress and its sources among oncology physicians and RNs in end‐of‐life decision‐making.	67 RNs	Mixed methods, survey, and open‐ended questions	RNs reported higher levels of moral distress primarily due to ethical uncertainties and involvement in decisions regarding life‐prolonging treatments. RNs’ moral distress was particularly high when patients had a low quality of life.
McAndrew & Hardin [39]	USA	To explore how RNs experience ethical conflict in the ICU and how organisational resources support them in addressing these conflicts.	80 RNs	Qualitative: open‐ended questions	Organisational resources have the potential to reduce moral distress. RNs identified ethics education, interprofessional dialogue, and greater involvement of RNs in ethical discussions as important strategies.
Robinson & Stinson [10]	USA	To determine how emergency RNs define moral distress, describe their experiences of it, and identify strategies to combat it.	8 RNs	Qualitative: interviews	Emergency RNs experience moral distress due to issues related to patient care, professional behaviour of other healthcare staff, and institutional constraints. These factors lead to feelings of frustration, helplessness, and burnout.
Morley et al. [40]	UK	To develop an empirically informed model for understanding and addressing moral distress among RNs.	21 RNs	Qualitative: interviews	Several compounding factors exacerbate/mitigate RNs ’ experiences of moral distress: epistemic injustice; conflict between one’s professional and personal responsibilities; ability to advocate and team dynamics.
Booth and Christian [41]	USA	To examine the surgical intensive care nurses′ experiences and cope with moral distress and how it affects their views of patient care.	21 RNs	Qualitative: interviews	Moral distress was associated with stress, poor communication, and lack of support undermined integrity and patient care.
Gan et al. [42]	China	To explore emergency nurses’ experiences of coping with moral distress using self‐efficacy theory.	17 RNs	Qualitative: semi‐structured interviews	Institutional constraints, resource shortages, and negative feedback challenged nurses’ coping confidence. Peer support, mentorship, and senior guidance were perceived as supportive, while emotional exhaustion and defensive coping were also reported.

The included studies were mapped across three review domains: experiences and sources of moral distress, coping strategies, and organisational responses or support. The mapping distinguished between domains explicitly examined by the studies, relevant findings reported, organisational approaches proposed by participants, and interventions that were implemented and evaluated. Several studies contributed evidence to more than one domain (Table 4).

**TABLE 4:** Mapping of included studies across review domains.

Study	Moral distress experiences/sources	Coping strategies	Organisational responses/support
Arends et al. [7]	E	—	—
Langley et al. [8]	E	E	—
Morley et al. [40]	E	F	—
Clark et al. [27]	E	—	—
Wolf et al. [31]	E	F	—
Choe et al. [36]	E	—	—
Mehlis et al. [38]	E	—	—
Demir et al. [11]	E	E	P
Asadi et al. [32]	E	—	—
Esmaeili et al. [33]	—	E	—
Vilela et al. [24]	E	—	—
Forozeiya et al. [13]	E	E	—
Hosseinpour and Keshmiri [25]	E	F	—
Lievrouw et al. [35]	—	E	—
Kiziltepe and Yilmaz [9]	E	F	P
McAndrew and Hardin [39]	E	F	E
Meziane et al. [28]	—	—	I
Ntseke et al. [37]	E	—	—
Robinson and Stinson [10]	E	E	—
Pavlish et al. [26]	E	—	E
Atli Özbaş et al. [34]	E	—	—
Miller et al. [29]	E	—	E
Tong et al. [30]	E	—	—
Booth and Christian [41]	E	E	—
Gan et al. [42]	E	E	P

*Note:* E = domain explicitly examined; F = relevant findings reported despite a different primary focus; P = organisational approaches proposed by study participants; I = implemented and evaluated intervention; — = no sufficient evidence reported for classification. Recommendations made solely by study authors were not counted as empirical evidence.

### 3.2. Moral Distress Experiences Among Registered Nurses in Adult Tertiary Care

#### 3.2.1. Patient Care–Related Challenges

A prominent concern across the literature was nurses’ limited autonomy and restricted involvement in treatment decisions [7, 24, 40]. Although advocacy was viewed as a core professional duty, hierarchical structures often positioned physicians as the primary decision‐makers, leaving nurses with minimal authority to influence care plans. This imbalance created ethical tension between nurses’ commitment to act in patients’ best interests and their inability to do so in practice [24]. The sense of being informed rather than consulted in life‐sustaining decisions was described as a recurring source of frustration and emotional exhaustion [8, 38]. In contrast, studies highlighted that collaborative decision‐making and recognition of nurses’ clinical insights mitigated distress by fostering shared accountability in patient care [40].

Ethical conflict was also prevalent in communication with patients and families, particularly when transparency was compromised or patient autonomy was overlooked. Nurses reported discomfort when expected to provide partial or misleading information or when witnessing unrealistic reassurance from physicians [7, 13, 33]. These practices, often intended to preserve hope, produced moral tension as nurses felt complicit in concealing the truth. Distress was especially pronounced in end‐of‐life situations, where decisions about aggressive or life‐prolonging treatments were made without adequate patient involvement [34, 38]. Such situations conflicted with principles of honesty and respect central to nursing ethics, deepening nurses’ sense of moral conflict.

A further recurring source of distress involved participating in care perceived as unsafe or futile. High workloads, inadequate staffing, and limited resources forced nurses to prioritise institutional demands over patient‐centred care, generating guilt and frustration [9, 31]. Nurses described emotional strain when required to perform interventions that prolonged suffering without improving outcomes, such as resuscitating terminally ill patients or maintaining life support for those with no prospect of recovery [8, 30, 39, 41].

#### 3.2.2. Interprofessional Communication and Collaboration

Deficiencies in communication between nurses, physicians, and other healthcare professionals were commonly reported as obstacles to ethical practice [9, 34]. Nurses described limited verbal and written communication within multidisciplinary teams, resulting in uncertainty about treatment plans and omissions in critical information [34]. These breakdowns were particularly problematic in end‐of‐life situations, where inadequate discussions regarding treatment limitations left nurses feeling responsible for decisions made without sufficient dialogue or clarity. In some cases, the inability to contact physicians for urgent support led to feelings of helplessness and frustration [9].

Distress was further intensified when physicians communicated vaguely with families, creating misunderstandings about patients’ prognoses and treatment outcomes [36]. Nurses often found themselves mediating between clinical realities and families’ expectations without the authority to provide full explanations, resulting in moral tension. Similarly, a lack of supportive feedback and frequent colleague criticism negatively affected nurses’ motivation and emotional well‐being, leaving them undervalued [27].

Inequitable power relations within interprofessional teams often place physicians in dominant positions, limiting nurses’ participation in decision‐making and diminishing their professional autonomy [24, 41]. Nurses described being expected to comply with medical directives despite having ethical concerns, particularly in complex care situations such as end‐of‐life decision‐making [8, 39]. Feeling overlooked or dismissed in these contexts eroded nurses’ confidence and moral agency, leading to emotional exhaustion and a diminished sense of professional worth [40].

#### 3.2.3. Emotional Well‐Being and Workplace Support

Moral distress was linked to inadequate emotional and organisational support. Nurses reported being expected to continue working after patient deaths without acknowledgement or debriefing, facing bullying from dominant colleagues, and being pressured to act against their ethical values [25, 37]. Lack of managerial recognition and unfair treatment further undermined morale [9, 34]. Distress also arose from fear of punitive responses when reporting unsafe or unethical practices, leading many nurses to remain silent despite recognising wrongdoing [32]. The absence of supportive leadership and psychological safety intensified emotional exhaustion and moral conflict.

#### 3.2.4. Administrative Challenges and Limitations

Resource scarcity and inadequate infrastructure were among the most frequently cited sources of moral distress [9, 31, 33, 34, 42]. Nurses described struggling to maintain safe care standards amid shortages of essential supplies, unreliable equipment, and understaffing. These constraints often forced them to compromise patient comfort and safety, contributing to guilt and frustration. Staffing deficits were particularly distressing, as they limited time for essential nursing care, increased fatigue, and reduced opportunities for compassionate interactions with patients [11, 36]. Broader systemic issues, including delayed administrative decisions and reduced services outside regular hours, further undermined the continuity and quality of care [32].

Institutional policies and administrative priorities strongly influenced nurses’ ethical practice and professional decision‐making. Several studies highlighted tensions between financial or operational goals and professional ethics [31, 36]. Cost‐driven decisions, such as premature discharges or patient transfers, were perceived as prioritising efficiency over patient welfare. Excessive documentation requirements and inefficient systems diverted time away from direct care, leaving nurses conflicted between administrative compliance and clinical responsibilities [11, 32, 39]. In some cases, policy directives promoting patient satisfaction led to overtreatment or ethically questionable decisions, further constraining nurses’ professional judgement [39].

High nurse–patient ratios further compounded these challenges. Many nurses described their workload as unmanageable, comparing the experience to working in a factory rather than delivering personalised care [11]. Inadequate staffing not only hindered quality care but also created an ongoing moral conflict between the desire to meet patient needs and the impossibility of doing so under current conditions [31, 34, 41].

Ambiguity surrounding professional roles and responsibilities was reflected in nurses being assigned tasks beyond their formal scope, particularly during emergencies or physician absences [9, 36]. This expansion of roles often placed them at legal and ethical risk, especially when adverse outcomes occurred, and accountability was directed towards nursing staff, leaving nurses feeling unsupported and vulnerable to blame [9].

Across these situations, moral distress arose when nurses recognised ethically appropriate care but were constrained by limited authority, inadequate resources, poor communication, workplace conditions, or organisational structures. Across the included studies, nurses reported responses ranging from active coping and support‐seeking to emotional distancing, acceptance, and withdrawal. The following sections therefore examine nurses’ coping strategies alongside the organisational resources and structures that may support or constrain these responses.

### 3.3. Coping Strategies Registered Nurses Employ to Mitigate Moral Distress

In response to the ethical, interpersonal, and organisational challenges described above, registered nurses employed a range of strategies to manage or adapt to moral distress. The included studies broadly characterised these strategies as adaptive or maladaptive, and their use appeared to be influenced by the broader clinical and organisational context and the availability of interpersonal or organisational support.

#### 3.3.1. Adaptive Coping Strategies

Effective strategies for managing moral distress include physical activity, yoga, relaxation practices, accessing psychological counselling, and engaging in staff debriefings (learning and professional growth), all of which have been recognised as beneficial in easing its impact [9, 31].

According to Lievrouw et al. [35], the coping approaches of registered nurses are shaped by their individual characteristics. Some adopt an internalising style, in which they take personal responsibility for ethical conflicts and engage in deep reflection. This approach encourages a sense of accountability and motivates them to improve their responses in future situations. Others employ more rational strategies, relying on their education, theoretical understanding, and evidence‐based practice to overcome ethical challenges. A further approach is experiential coping, where nurses draw on previous experiences and rely on peer support through group discussions and informal conversations. This not only provides emotional relief but also allows them to share practical insights with colleagues [35].

Esmaeili et al. [33] expanded on these findings by noting that many nurses actively built their professional knowledge as a coping resource. They consulted senior colleagues, reviewed relevant literature, and participated in workshops, training programmes, and ward rounds. Through this commitment to continuous learning, they gained reassurance that their decisions were both informed and appropriate, which strengthened their confidence and capacity to manage future ethical dilemmas effectively.

Reflection has been recognised as an effective approach to managing moral distress. Demir et al. [11] advised incorporating regular reflection sessions, both in small groups and larger settings, to create structured spaces where nurses can process emotions and challenges. Participants in their study emphasised the value of speaking with individuals familiar with the demands of nursing practice, as this ensured more genuine and effective support.

Another coping method, often described as rejuvenation, involves temporarily stepping away from the clinical environment to restore energy and motivation. Nurses reported achieving this through quality time with family and close friends, engaging in prayer or spiritual practices, and confiding in trusted people, whether relatives or colleagues. These activities were seen to replenish emotional strength and prepare them to confront future ethical difficulties [33]. Experienced nurses often rely on resilience and adaptability to cope with moral distress, describing the process metaphorically as being “like grass in the wind” ([13], p. 26). While challenges persist, they confront distress by drawing on their professional expertise, knowledge, and experience to manage complex situations. Instead of remaining passive, they take proactive steps by advocating for patients, initiating conversations with healthcare providers and caregivers, and arranging family meetings. Such actions reflect their determination to uphold professional values while adjusting to difficult circumstances [13].

Teamwork was also identified as a vital resource for easing moral distress. Demir et al. [11] emphasised the significance of collaboration and mutual encouragement, particularly in guiding newly recruited staff and avoiding criticism of stressed colleagues. Building a supportive atmosphere by sharing positivity, celebrating small achievements, and motivating one another was considered key to sustaining morale. The use of checklists to document progress further enhanced this effect by giving staff a tangible sense of accomplishment, thereby reducing distress at both individual and team levels [11]. Similarly, Gan et al. [42] found that peer discussion, senior guidance, and reassurance supported nurses’ confidence in managing morally distressing situations.

Compensation refers to viewing moral distress as something that cannot be fully resolved and addressing it by concentrating on different aspects of care. Nurses may spend extra time with patients, aim to deliver the highest possible quality of care, or even ask unconscious patients for forgiveness as a way to find emotional relief [33]. Others compensate by reporting errors or negligence, thereby contributing to corrective measures and preventing future incidents. This strategy helps them cope by fostering accountability and restoring a sense of emotional balance [33].

Turning towards, on the other hand, involves openly recognising the presence of moral distress and actively building support through professional relationships. This method, commonly observed among newer nurses, highlights the importance of creating trusting bonds with more experienced colleagues who can offer empathy, affirmation, and practical guidance when facing ethical challenges [13, 33].

Some nurses turned to their faith, using prayer to ease emotional pressure and drawing comfort from the belief that patient outcomes were guided by God’s will. This outlook helped them accept and rationalise the difficulties associated with end‐of‐life care [8]. Additional strategies involved temporarily disconnecting from work, such as taking time off to regain balance or sharing experiences with colleagues to process emotions [8]. Another approach, described as “turning away”, involved deliberately creating distance from moral distress through distractions or concentrating on routine tasks. Examples included short breaks, reading, or going outdoors. These activities enabled nurses to shift their attention briefly, giving their minds a chance to rest and recover [13].

#### 3.3.2. Maladaptive Coping Strategies

Registered nurses, at times, relied on maladaptive coping methods to manage the emotional strain of moral distress. These included behaviours such as drinking alcohol, overeating, taking medication, or disregarding their feelings [9, 31]. Some resorted to more harmful actions, including suppressing emotions, relying on denial to block distress, or even turning to drug use after especially challenging shifts [10]. Although such strategies enabled them to remain functional in the moment, they often resulted in emotional withdrawal and unresolved distress, producing harmful consequences for both personal well‐being and professional practice [10].

Other coping patterns involved acceptance and silence. In these cases, senior nurses sometimes ignored violations of professional standards, treated ethical problems as part of the system, and chose not to respond to continue functioning within the workplace environment [25].

Withdrawal was identified as one strategy that nurses attempted to use to manage repeated exposure to moral distress. In this approach, they deliberately created emotional distance by concentrating on technical duties, which helped suppress empathy and limit their connection to the ethical situation [40]. Gan et al. [42] also reported defensive coping and emotional detachment following negative feedback and repeated morally distressing experiences. Yet this method rarely provided complete relief, as their obligation to fulfil nursing responsibilities maintained some degree of involvement, often resulting in additional guilt and a sense of moral shortcoming [40].

At times, nurses faced ethical challenges by acknowledging their lack of influence and accepting that they had little power to change difficult circumstances [39]. Lievrouw et al. [35] described this as an externalising style, where responsibility was shifted away from the individual, and moral distress was perceived as something outside their control. This perspective allowed them to safeguard their emotional well‐being by avoiding deeper engagement with distressing experiences.

Esmaeili et al. [33] identified several coping responses considered less favourable. Some nurses developed indifference, shielding themselves from distress by staying silent and refraining from action in ethically difficult situations when support was lacking. Others used escape, which meant avoiding distress through resignation, department transfer, or shifting responsibility by reporting concerns to supervisors. A further approach, concealment, involved altering or omitting documentation to protect themselves or patients when they judged certain interventions as harmful or inappropriate.

Across the included studies, coping strategies were generally described as ways of managing or adapting to moral distress rather than as responses specific to particular sources of moral distress. Their perceived usefulness varied, with some strategies supporting reflection, resilience, and continued professional engagement, while others provided temporary relief but were associated with withdrawal, avoidance, or unresolved distress. The organisational context also appeared important in shaping nurses’ capacity to cope, as examined in the following section.

#### 3.3.3. Relationship Between Moral Distress Contexts and Coping Responses

Across the included studies, nurses used a range of adaptive and maladaptive strategies to cope with moral distress [13, 31, 33, 35]. However, most studies examined the sources or experiences of moral distress and coping strategies as related but distinct phenomena and did not systematically examine whether particular organisational, interpersonal, or ethical stressors led nurses to select specific coping strategies. Consequently, direct relationships between individual sources of moral distress and particular coping responses could not be consistently established across the evidence base. Nevertheless, some studies indicated that coping was influenced by the broader clinical and organisational context, including professional experience, peer and managerial support, workplace hierarchy, communication, and nurses’ perceived ability to influence the situation [13, 33, 35, 39].

### 3.4. The Organisational Roles and Functions to Address Moral Distress Among RNs

While nurses used individual and interpersonal strategies to manage moral distress, several studies emphasised that coping occurred within an organisational context [40]. The included studies identified organisational resources, leadership practices, workplace conditions, education, and collaborative structures as factors that could support nurses in managing moral distress or addressing its contributing conditions [9, 11, 26, 39].

Organisational responses identified in the included studies operated at different levels. Some approaches, such as counselling, structured debriefing, and facilitated reflective practice, primarily supported nurses’ individual coping but depended on organisational provision. Others, including leadership practices, staffing and resource allocation, collaborative decision‐making, and ethics support structures, focused on the organisational conditions associated with moral distress.

#### 3.4.1. Support and Resource Allocation

Stress management resources, debriefing opportunities, and end‐of‐life care training were identified as potential organisational strategies for supporting nurses experiencing moral distress, particularly those with limited experience [8, 10]. Langley et al. [8] also highlighted emotional support and professional counselling as potentially valuable resources for reflection, self‐care, and managing moral distress. Furthermore, Tong et al. [30] reported that nurses who felt better prepared to answer families’ questions about end‐of‐life care experienced lower levels of moral distress. This finding highlights the potential importance of preparation for communication with families in ethically challenging clinical situations.

#### 3.4.2. Enhancing Workplace Conditions

Kiziltepe and Yilmaz [9] identified clearer professional roles and responsibilities, appropriate clinical placements, improved staffing ratios, and greater managerial engagement with frontline challenges as suggested approaches to improving nurses’ working conditions. In Demir et al. [11], nurses proposed higher wages as a potential approach to attracting additional staff and improving staffing levels. Pavlish et al. [26] identified regular discussions about ethical concerns involving patients, families, and healthcare teams as an important approach to promoting communication and addressing moral distress.

#### 3.4.3. Nursing Leadership in Shaping Practice

Nursing leadership emerged as an important source of organisational support. Nurses in Demir et al. [11] emphasised the need for greater managerial awareness of frontline challenges and identified recognition and encouragement from senior management as important for morale. Miller et al. [29] reported that nursing leaders addressed moral distress through staff advocacy, communication with families and healthcare teams, opportunities for emotional support and decompression, and maintaining a visible presence within clinical units.

Pavlish et al. [26] identified several leadership approaches to addressing moral distress, including being accessible, listening to staff concerns, advocating for nurses, and promoting open communication. The study also highlighted ethics education, self‐care resources, teamwork, and opportunities for debriefing as potential ways to strengthen organisational support for nurses experiencing ethically difficult situations.

#### 3.4.4. Enhancing Collaborative Practice

The included studies identified interprofessional communication and collaboration as important organisational approaches to addressing moral distress. McAndrew and Hardin [39] and Pavlish et al. [26] highlighted transparent communication, collaborative problem‐solving, and interdisciplinary discussions as ways to support nurses in ethically challenging situations. In Demir et al. [11], nurses also recommended delegating non‐nursing duties, such as patient transport, to reduce workload pressures and allow greater attention to nursing responsibilities. These findings reflect nurses’ and nursing leaders’ perspectives on potentially supportive organisational practices rather than demonstrating their effectiveness in reducing moral distress.

#### 3.4.5. Learning and Professional Growth

Ethics education, reflective practice, and professional development were identified as potential organisational resources for addressing moral distress. McAndrew and Hardin [39] highlighted case‐based ethics education, opportunities for reflection, and discussions with colleagues as approaches that nurses considered valuable when managing ethical challenges. Demir et al. [11] also emphasised professional development and strengthening nurses’ competencies as important areas for organisational support. Encouraging reflection on personal experiences and promoting teamwork‐oriented discussions with colleagues were also highlighted as valuable strategies for guiding nurses through ethical challenges [39]. Moreover, focusing on building nurses’ competencies not only enhances patient safety but also supports their individual growth and career advancement [11]. Gan et al. [42] also reported nurses’ suggestions for ethics training, mentorship, and psychologically informed support to strengthen coping confidence.

Meziane et al. [28] evaluated a reflective practice intervention among nurses providing end‐of‐life care. Half of the participants (*n* = 8) reported improvements in their practice, while nearly one‐third (*n* = 5) reported increased professional confidence, enabling them to question certain medical practices. Although three nurses who had previously considered leaving their positions changed their minds following the intervention, the overall change in intention to leave was not statistically significant. Similarly, the intervention did not produce a statistically significant reduction in mean moral distress scores (*p* = 0.62). These findings suggest that reflective practice may support nurses’ professional confidence and perceived ability to manage ethical challenges, although its effectiveness in reducing moral distress remains uncertain.

#### 3.4.6. Ethical Support and Family Inclusion

McAndrew and Hardin [39] identified nurses’ involvement in ethics consultations, opportunities for postcase debriefing, and access to ethics‐related support as important organisational resources for addressing moral distress. Nurses also expressed interest in gaining a better understanding of organisational ethics committees and receiving support following ethically challenging situations.

Nurses also highlighted the importance of participating in ethical discussions to ensure that nursing perspectives were considered in care decisions [39]. The study further identified patient and family education, written information about ethical issues, and regular family meetings as proposed approaches to improving communication and supporting ethical decision‐making.

Overall, the included studies identified a range of organisational approaches to addressing moral distress, including supportive leadership, improved communication, ethics education, reflective practice, and access to emotional support. However, much of the available evidence reflected nurses’ and nursing leaders’ reported experiences, perceived needs, and proposed strategies, rather than evaluations of implemented interventions. This limited intervention‐focused evidence highlights a gap in understanding how effective organisational approaches are at reducing moral distress among registered nurses in adult tertiary care settings.

## 4. Discussion

This review synthesises evidence on nurses’ experiences of moral distress, coping strategies, and organisational responses within adult tertiary care settings. The findings highlight how organisational, interpersonal, and ethical conditions intersect in nurses’ experiences of moral distress, particularly when resource limitations, power imbalances, poor communication, or exclusion from decision‐making constrain their ability to provide care consistent with their professional values. Nurses’ coping responses were also shaped by professional experience, perceived agency, team relationships, and organisational support. Together, these findings emphasise the importance of considering moral distress, coping strategies, and organisational responses as interconnected dimensions.

### 4.1. Organisational and Contextual Determinants of Moral Distress

A central finding was the prominent role of organisational conditions in generating and sustaining moral distress. Staffing and resource limitations, organisational priorities, hierarchical power relations, ineffective communication, and limited nursing involvement in ethical decisions constrained nurses’ moral agency [24, 31, 39, 40]. Wolf et al. [31], for example, characterised moral distress in emergency nursing as largely environment‐driven, while McAndrew and Hardin [39] showed how organisational culture and the marginalisation of nurses during ethical conflict contributed to distress. These findings suggest that moral distress cannot be addressed solely by strengthening nurses’ ability to manage its consequences; modifiable organisational conditions also require attention.

Cultural and professional contexts may further shape which situations nurses experience as morally troubling and how they respond. Expectations surrounding professional hierarchy, truth‐telling, family involvement, and end‐of‐life decision‐making may affect nurses’ ethical agency. Organisational responses should therefore be context‐sensitive while preserving nurses’ ability to raise concerns and participate meaningfully in decisions.

Some included studies extended the concept of moral distress beyond the classic constraint‐based definition, in which individuals recognise an ethically appropriate action but are constrained from acting accordingly [43]. Some studies reported emotionally challenging experiences such as patient death or profound patient suffering as sources of moral distress [8, 38]. However, these findings did not always clearly articulate an ethical constraint. This may blur the distinction between moral distress and emotional or occupational distress.

### 4.2. Contextual Influences on Coping

The included studies identified a range of coping strategies, including reflection, seeking peer support, professional learning, patient advocacy, emotional distancing, and avoidance [13, 31, 33, 35]. While some strategies supported nurses’ continued engagement with ethical challenges, others involved withdrawing from distressing situations or accepting circumstances they felt unable to change. This variation highlights the complexity of nurses’ responses to moral distress and suggests that coping strategies may not always address the underlying ethical or organisational challenges.

Coping appeared to be context‐dependent rather than reflecting a simple relationship between particular stressors and specific strategies. Lievrouw et al. [35] reported variations in coping associated with professional experience and team culture, while Esmaeili et al. [33] identified managerial support, communication, hierarchy, fear of reprimand, and powerlessness as influences on nurses’ preferred coping strategies. Forozeiya et al. [13] also highlighted the importance of support from experienced colleagues. These findings suggest that coping may be influenced by nurses’ perceived agency, professional experience, and practice environment.

However, most included studies did not systematically examine direct relationships between specific stressors and coping strategies. Therefore, although the literature identified both sources of moral distress and coping responses, it remains unclear how particular stressors influence the selection of specific strategies. Future research should examine how professional experience, perceived controllability, team relationships, and organisational support shape coping responses over time.

### 4.3. Organisational Interventions, Ethical Climate, and Nurse Leadership

A key implication of this review is the distinction between individual coping and organisational intervention. Individual strategies such as reflection, self‐care, spiritual coping, emotional regulation, or informal support primarily help nurses manage their response to distress. Organisational interventions may instead provide formal support or modify the conditions contributing to moral distress. Consequently, individual coping alone cannot address persistent understaffing, inadequate resources, exclusion from decision‐making, ineffective communication, or restrictive hierarchies.

Counselling, structured debriefing, facilitated reflective practice and formal peer support may be understood as organisation‐enabled coping resources, as they primarily support nurses’ individual responses to moral distress while depending on organisational provision. In contrast, approaches such as staffing and resource allocation, leadership practices, and shared decision‐making structures aim to address the organisational conditions contributing to moral distress. Ethics consultation may operate at both levels by supporting individual nurses in ethically challenging situations while also providing a formal organisational mechanism for addressing ethical concerns. McAndrew and Hardin [39] identified ethics education, interprofessional dialogue, and greater nursing involvement as potentially important organisational resources, while Pavlish et al. [26] highlighted leadership actions including advocacy, debriefing, collaboration, and ethics consultation.

However, much of the evidence concerning organisational responses reflected nurses’ and nursing leaders’ perceptions of supportive practices rather than evaluations of implemented interventions. Meziane et al. [28] evaluated a reflective practice intervention and reported perceived improvements in professional confidence and clinical practice, but no statistically significant reduction in moral distress. This illustrates the distinction between perceived benefits and demonstrated effectiveness and highlights the limited intervention‐focused evidence currently available.

These findings also emphasise the importance of ethical climate and nurse leadership. Environments in which nurses can voice ethical concerns, participate in decisions, and access support may strengthen nurses’ ability to act on ethical concerns, whereas hierarchical or punitive environments may reinforce silence and powerlessness. This interpretation aligns with broader evidence showing an inverse relationship between a favourable ethical climate and moral distress [44]. Nurse leaders therefore have a role not only in supporting nurses after distressing events but also in shaping conditions that promote psychological safety, ethical dialogue, professional voice, and responsive organisational action.

Overall, the evidence suggests that individual coping and organisational responses address different but interconnected aspects of moral distress. While coping strategies may help nurses manage its emotional and professional consequences, organisational approaches may address the conditions that contribute to distress. However, the limited evaluation of organisational interventions means that their effectiveness remains uncertain. These findings highlight the importance of considering both individual and organisational approaches when developing strategies to address moral distress.

### 4.4. Implications for Nursing Management

The findings have important implications for nursing management and organisational policy. Limited nursing involvement in decision‐making, inadequate staffing and resources, hierarchical relationships, and poor communication were recurring contributors to moral distress [7, 11, 31, 34]. Organisational and policy responses should therefore strengthen nurses’ participation in ethical and clinical decision‐making, improve staffing and resource provision, and establish safe mechanisms for raising ethical concerns. Ethics consultation, structured debriefing, counselling, and other formal support mechanisms may also provide nurses with opportunities to process morally challenging experiences [26].

Nurse leaders have a particularly important role in creating supportive ethical practice environments. Promoting open communication, interdisciplinary collaboration, visible leadership, and nonpunitive responses to ethical concerns may strengthen nurses’ professional voice and ability to address moral challenges [11, 26, 39]. Clarifying professional roles and encouraging nurses’ participation in team and family discussions may further reduce the sense of powerlessness associated with moral distress. These approaches recognise that responsibility for managing moral distress should not rest solely with individual nurses but should also involve organisational structures and leadership practices.

Education should complement these organisational measures by strengthening nurses’ ethical reasoning, communication, reflection, and capacity to respond to morally complex situations. Case‐based ethics education, reflective practice, mentorship, and continuing professional development may help nurses recognise and respond to ethical challenges [33, 40]. Preparation for communication with patients and families may be particularly relevant in end‐of‐life care, where greater preparedness has been associated with lower moral distress [30]. Educational approaches should also recognise cultural and contextual differences in how moral distress is experienced and managed. However, most of these approaches were proposed or reported as supportive rather than formally evaluated for their effectiveness in reducing moral distress.

### 4.5. Implications for Future Research

The evidence base remains limited regarding the effectiveness of organisational interventions for moral distress. Although counselling, structured debriefing, reflective practice, ethics support, and supportive leadership were identified as potentially valuable resources, further research is needed to evaluate their effectiveness, sustainability, and longer term outcomes using robust intervention designs. Future studies should also examine how professional experience, perceived control, team relationships, and organisational support influence coping and moral resilience over time. Longitudinal and intervention‐focused research may help clarify how cumulative exposure to moral distress develops across nurses’ careers and which organisational conditions best support constructive coping and sustained ethical engagement. Another overlooked area is how professional experience influences coping with moral distress. Evidence suggests that nurses’ responses may vary with professional experience and access to experienced colleagues and workplace support [13, 33]. However, none of the included studies focused specifically on newly graduated registered nurses. This gap matters because early‐career nurses may have fewer experiential resources and rely more on senior colleagues to interpret and respond to morally distressing situations. Research specifically on NGRNs is therefore needed to understand how they experience and manage moral distress during the transition to professional practice and which forms of organisational support are most relevant during this period. A recent study protocol also identified NGRNs as a population that requires focused investigation of moral distress in adult tertiary care settings [45]. Future research should also examine the relationships between organisational conditions, moral distress, coping strategies, and related outcomes, particularly through longitudinal and intervention studies.

### 4.6. Strengths and Limitations

This review synthesises international evidence on moral distress among registered nurses in adult tertiary care settings, providing insights into its ethical, organisational, and contextual dimensions. However, restricting inclusion to English‐language, peer‐reviewed publications may have excluded relevant evidence. No formal quality appraisal was undertaken, consistent with JBI guidance for scoping reviews. Variation in clinical specialities, participant characteristics, healthcare contexts, study designs, and conceptualisations of moral distress may affect the interpretation and applicability of the findings. Furthermore, no studies specifically focused on newly graduated registered nurses were identified, highlighting an evidence gap. Despite these limitations, the review provides a foundation for future research and organisational practice.

## 5. Conclusion

Moral distress among registered nurses in adult tertiary care reflects the complex interaction between ethical challenges in clinical practice and the organisational conditions that influence nurses’ capacity to act on their professional values. The evidence synthesised in this review demonstrates that moral distress has wide‐ranging implications for nurses’ well‐being, the quality and consistency of patient care, and the stability of the nursing workforce. Efforts to mitigate moral distress must therefore extend beyond individual coping, recognising the crucial role of supportive organisational cultures, adequate staffing, shared decision‐making structures, and strong clinical leadership. The included studies identified ethics support, reflective practice, and ongoing education as potentially valuable approaches to supporting nurses experiencing moral distress, although evidence of their effectiveness remains limited. At a system level, organisational approaches that promote nurses’ involvement in decision‐making, open ethical dialogue, and attention to structural barriers may help create more supportive ethical practice environments. Given the limited evidence on effective organisational interventions, further research is needed to evaluate strategies that can meaningfully reduce moral distress and enhance ethical practice within complex tertiary care settings.

## Funding

This review did not receive a specific grant from any funding agency in the public, commercial, or not‐for‐profit sectors. This review forms part of the first author’s doctoral research, which is funded by a scholarship from the Ministry of Education, Saudi Arabia.

## Conflicts of Interest

The authors declare no conflicts of interest.

## Data Availability

No new primary data were collected during this scoping review. All data supporting the findings were derived from published studies and are presented in the manuscript.

## References

[1] Dodek P. M. , Wong H. , Norena M. et al., Moral Distress in Intensive Care Unit Professionals is Associated With Profession, Age, and Years of Experience, Journal of Critical Care. (2016) 31, no. 1, 178–182, 10.1016/j.jcrc.2015.10.011. 26596697

[2] Bagshaw M. , Bellows M. , Dhaliwal J. , Johnson-Coyle L. , Opgenorth D. , and Richardson-Carr S. , Moral Distress and Burnout Among Healthcare Providers in a Cardiovascular Intensive Care, Canadian Journal of Cardiology. (2016) 32, no. 10, 10.1016/j.cjca.2016.07.434. 29786979

[3] Giannetta N. , Sergi R. , Villa G. et al., Levels of Moral Distress Among Health Care Professionals Working in Hospital and Community Settings: A Cross-Sectional Study, Healthcare (Basel). (2021) 9, no. 12, 10.3390/healthcare9121673. PMC8701919 34946401

[4] Salari N. , Shohaimi S. , Khaledi-Paveh B. , Kazeminia M. , Bazrafshan M. R. , and Mohammadi M. , The Severity of Moral Distress in Nurses: A Systematic Review and meta-analysis, Philosophy, Ethics, and Humanities in Medicine. (2022) 17, no. 1, 10.1186/s13010-022-00126-0. PMC9644548 36348378

[5] Caram C. da S. , Rezende L. C. , Fonseca M. P. et al., Strategies for Coping with Distress Adopted by Nurses in Tertiary Care: A Scoping Review, Texto & Contexto-Enfermagem. (2022) 31, no. 8, 10.1590/1980-265x-tce-2021-0159.

[6] Borhani F. , Abbaszadeh A. , Mohamadi E. , Ghasemi E. , and Hoseinabad-Farahani M. J. , Moral Sensitivity and Moral Distress in Iranian Critical Care Nurses, Nursing Ethics. (2015) 24, no. 4, 474–482, 10.1177/0969733015604700. 26419438

[7] Arends S. A. M. , Steenbergen M. , Thodé M. , Francke A. L. , and Jongerden I. P. , Moral Distress Among Nurses Involved in life-prolonging Treatments in Patients With a Short Life Expectancy: A Qualitative Interview Study, Patient Education and Counseling. (2022) 105, no. 7, 2531–2536, 10.1016/j.pec.2022.01.017. 35123835

[8] Langley G. C. , Kisorio L. , and Schmollgruber S. , Moral Distress Experienced by Intensive Care Nurses, Southern African Journal of Critical Care. (2015) 31, no. 2, 10.7196/SAJCC.2015.v31i2.235.

[9] Kiziltepe S. and Yilmaz Ş. , Turkish Intensive Care Nurses’ Experiences of Moral Distress: A Qualitative Study, Turkiye Klinikleri Journal of Nursing Sciences. (2022) 14, no. 3, 798–806, 10.5336/nurses.2021-87920.

[10] Robinson R. and Stinson C. K. , Moral Distress: A Qualitative Study of Emergency Nurses, Dimensions of Critical Care Nursing. (2016) 35, no. 4, 235–240, 10.1097/DCC.0000000000000185. 27258962

[11] Demir M. , Håkansson E. , and Drott J. , Nurses’ Experiences of Moral Distress and How it Affects Daily Work in Surgical Care—A Qualitative Study, Journal of Advanced Nursing. (2024) 80, no. 5, 2080–2090, 10.1111/jan.15966. 37975326

[12] Epstein E. G. and Delgado S. , Understanding and Addressing Moral Distress, OJIN: Online Journal of Issues in Nursing. (2010) 15, no. 3, 10.3912/OJIN.Vol15No03Man01.

[13] Forozeiya D. , Vanderspank-Wright B. , Bourbonnais F. F. , Moreau D. , and Wright D. K. , Coping with Moral Distress – the Experiences of Intensive Care Nurses: An Interpretive Descriptive Study, Intensive and Critical Care Nursing. (2019) 53, 23–29, 10.1016/j.iccn.2019.03.002. 30948283

[14] Bolton S. , Willis E. , and Byrne A. L. , The Role of Leadership in Supporting and Mitigating Moral Distress in Nursing: A Scoping Review, Journal of Nursing Management. (2025) 2025, 10.1155/jonm/5443770. PMC12370389 40851909

[15] Arad M. , Amini M. , Fattahi Z. , Seidi J. , and Khaninezhad L. , Moral Distress Among Nurses: An Umbrella Review of Its Prevalence, Severity, Causes, and Consequences, BMC Nursing. (2026) 25, 10.1186/s12912-026-04887-3. PMC13501555 42288828

[16] Sobhanifar F. , Mirhosseini S. , Ghorbani Vajargah P. , Taheri-Ezbarami Z. , and Emami Sigaroudi A. , Interventions Addressing Moral Distress Among Nurses: A Systematic Review, Nursing Ethics. (2026) 10.1177/09697330261490879. 42859878

[17] Peters M. D. J. , Godfrey C. , McInerney P. et al., Best Practice Guidance and Reporting Items for the Development of Scoping Review Protocols, JBI Evidence Synthesis, Advance online publication. (2022) 20, no. 4, 953–968, 10.11124/JBIES-21-00242. 35102103

[18] Tricco A. C. , Lillie E. , Zarin W. et al., PRISMA Extension for Scoping Reviews (PRISMA-ScR): Checklist and Explanation, Annals of Internal Medicine. (2018) 169, no. 7, 467–473, 10.7326/M18-0850. 30178033

[19] Huo M. , Zhao B. , Li Y. , and Li J. , Evidence-Based Practice Dynamic Capabilities: A Concept Derivation and Analysis, Annals of Translational Medicine. (2022) 10, no. 1, 10.21037/atm-21-6506. PMC8825542 35242867

[20] DeMars M. M. and Perruso C. , Mesh and Text-word Search Strategies: Precision, Recall, and Their Implications for Library Instruction, Journal of the Medical Library Association. (2022) 110, no. 1, 23–33, 10.5195/jmla.2022.1283. PMC8830400 35210959

[21] Aveyard H. , Doing a Literature Review in Health and Social Care: A Practical Guide, 2023, 5th edition, Open University Press, McGraw-Hill, Maidenhead.

[22] Oermann M. H. , Wrigley J. , Nicoll L. H. , Ledbetter L. S. , Carter-Templeton H. , and Edie A. H. , Integrity of Databases for Literature Searches in Nursing, Advances in Nursing Science. (2020) 44, no. 2, 102–110, 10.1097/ANS.0000000000000349. PMC8115732 33315590

[23] Braun V. and Clarke V. , Thematic Analysis: A Practical Guide, 2022, SAGE Publications Ltd, London.

[24] Vilela G. S. , Ferraz C. M. L. C. , Moreira D. A. , and Brito M. J. M. , Ethics and Moral Distress Expressions in Intensive Care Nursing Practice, Acta Paulista de Enfermagem. (2021) 34, 10.37689/acta-ape/2021AO01661.

[25] Hosseinpour A. and Keshmiri F. , Inductive Process of Moral Distress Development in Viewpoints from Surgical Nurses: A mixed-method Study, BMC Nursing. (2024) 23, no. 1, 10.1186/s12912-024-01786-3. PMC10956303 38515072

[26] Pavlish C. , Brown-Saltzman K. , So L. , and Wong J. , SUPPORT: An Evidence-based Model for Leaders Addressing Moral Distress, The Journal of Nursing Administration: The Journal of Nursing Administration. (2016) 46, no. 6, 313–320, 10.1097/nna.0000000000000351. 27214334

[27] Clark P. , Hulse B. , and Polivka B. J. , Resilience, Moral Distress, and Job Satisfaction Driving Engagement in Emergency Department Nurses: A Qualitative Analysis, The Journal of Nursing Administration. (2022) 52, no. 2, 112–117, 10.1097/NNA.0000000000001111. 35060953

[28] Meziane D. , Ramirez-Garcia M. P. , and Fortin M.-L. , A Reflective Practice Intervention to Act on the Moral Distress of Nurses Providing end-of-life Care on Acute Care Units, International Journal of Palliative Nursing. (2018) 24, no. 9, 444–451, 10.12968/ijpn.2018.24.9.444. 30260298

[29] Miller P. H. , Epstein E. G. , Smith T. B. , Welch T. D. , Smith M. , and Bail J. R. , Critical Care Nurse Leaders Addressing Moral Distress: A Qualitative Study, Nursing in Critical Care. (2024) 29, no. 4, 835–838, 10.1111/nicc.13045. 38400568

[30] Tong H. H. , Creutzfeldt C. J. , Hicks K. G. , Kross E. K. , Sharma R. K. , and Jennerich A. L. , Questions from Family Members During the Dying Process and Moral Distress Experienced by ICU Nurses, Journal of Pain and Symptom Management. (2024) 67, no. 5, 402–410, 10.1016/j.jpainsymman.2024.01.041. 38342474

[31] Wolf L. A. , Perhats C. , Delao A. M. , Clark P. R. , Edwards C. , and Frankenberger W. D. , It’s a Burden You Carry: Describing Moral Distress in Emergency Nursing, Journal of Emergency Nursing. (2016) 42, no. 1, 37–46, 10.1016/j.jen.2015.08.008. 26431742

[32] Asadi N. , Royani Z. , Maazallahi M. , and Salmani F. , Being Torn by Inevitable Moral Dilemma: Experiences of ICU Nurses, BMC Medical Ethics. (2021) 22, no. 1, 10.1186/s12910-021-00727-y. PMC8638377 34847926

[33] Esmaeili M. , Navidhamidi M. , and Varasteh S. , Coping Strategies of Intensive Care Unit Nurses Reducing Moral Distress: A Content Analysis Study, Nursing Ethics. (2024) 31, no. 8, 1586–1599, 10.1177/09697330241246089. 38628067

[34] Atli Özbaş A. , Kovanci M. S. , and Köken A. H. , Moral Distress in Oncology Nurses: A Qualitative Study, European Journal of Oncology Nursing. (2021) 54, 10.1016/j.ejon.2021.102038. 34601227

[35] Lievrouw A. , Vanheule S. , Deveugele M. et al., Coping with Moral Distress in Oncology Practice: Nurse and Physician Strategies, Oncology Nursing Forum. (2016) 43, no. 4, 505–512, 10.1188/16.onf.505-512. 27314193

[36] Choe K. , Kang H. , and Park Y. , Moral Distress in Critical Care Nurses: A Phenomenological Study, Journal of Advanced Nursing. (2015) 71, no. 7, 1684–1693, 10.1111/jan.12638. 25688835

[37] Ntseke S. , Coetzee I. , and Heyns T. , Moral Distress Among Critical Care Nurses when Executing do-not-resuscitate (DNR) Orders in a Public Critical Care Unit in Gauteng, The Southern African journal of critical care. (2023) 39, no. 2, 49–53, 10.7196/SAJCC.2023.v39i2.511. PMC10399545 37547770

[38] Mehlis K. , Bierwirth E. , Laryionava K. et al., High Prevalence of Moral Distress Reported by Oncologists and Oncology Nurses in end-of-life Decision Making, Psycho-Oncology. (2018) 27, no. 12, 2733–2739, 10.1002/pon.4868. 30156350

[39] McAndrew N. S. and Hardin J. B. , Giving Nurses a Voice During Ethical Conflict in the Intensive Care Unit, Nursing Ethics. (2020) 27, no. 8, 1631–1644, 10.1177/0969733020934148. 32662744

[40] Morley G. , Bradbury‐Jones C. , and Ives J. , The Moral Distress Model: An Empirically Informed Guide for Moral Distress Interventions, Journal of Clinical Nursing. (2022) 31, no. 9–10, 1309–1326, 10.1111/jocn.15988. 34423483

[41] Booth A. T. and Christian B. J. , Surgical Intensive Care Unit Nurses’ Coping with Moral Distress and Moral Residue: A Descriptive Qualitative Approach, Dimensions of Critical Care Nursing. (2024) 43, no. 6, 298–305, 10.1097/DCC.0000000000000665. 41401120

[42] Gan L. , Huang L. , Wu S. , Li H. , Mao Z. , and Deng Q. , A Qualitative Study on Emergency Department Nurses’ Experiences of Coping with Moral Distress Based on self-efficacy Theory, Frontiers in Public Health. (2026) 14, 10.3389/fpubh.2026.1849971. PMC13377816 42491570

[43] Epstein E. G. and Hamric A. B. , Moral Distress, Moral Residue, and the Crescendo Effect, Journal of Clinical Ethics. (2009) 20, no. 4, 330–342, 10.1086/JCE200920406. 20120853

[44] Xue K. , Shang J. , Yang C. , Pan L. , Shi H. , and Zeng Y. , Nurses’ Moral Distress and Ethical Climate: A Systematic Review and meta-analysis, Nursing Ethics. (2025) 32, no. 7, 1981–1997, 10.1177/09697330251350384. 40550472

[45] Alfaifi H. , Brown M. , McVeigh C. , and Marsh L. , Exploring the Views and Experiences of Moral Distress Among Newly Graduated Registered Nurses in Saudi Arabia: A Sequential Explanatory mixed-methods Study Protocol, Nursing Reports. (2026) 16, no. 7, 10.3390/nursrep16070240. PMC13416062 42506027

